# Experimental Study on the Effects of Matric Suction on Shear Properties of Polypropylene Fiber Reinforced Unsaturated Clay

**DOI:** 10.3390/ma15228223

**Published:** 2022-11-19

**Authors:** Ruiqian Wu, Guang Yang, Shaohe Li, Qichen Xiang

**Affiliations:** 1 College of Civil Engineering, Shaoxing University, Shaoxing 312000, China; 2 School of Architecture and Engineering, Zhejiang Industry Polytechnic College, Shaoxing 312000, China

**Keywords:** unsaturated clay, matric suction, polypropylene fiber, shear properties, behavior of unsaturated soils

## Abstract

Matric suction has an important effect on the behavior of unsaturated soils, and polypropylene fibers are often used to improve soil. In order to probe into the mechanism of matric suction and fiber reinforcement, triaxial shear tests are carried out with changing matric suction and net confining pressure. Unsaturated clay in the Shaoxing section of East Zhejiang Grand Canal is selected with polypropylene fiber as a reinforcement material in the tests. The results show that the total cohesion intercept and effective internal friction angle of soil increase with the increase in matric suction, while the adsorption internal friction angle decreases gradually. Similarly, the contribution of matric suction to shear strength decreases. The total cohesion intercept is more sensitive to matric suction. As the length of fiber is 12 mm, the shear strength parameters of soil will be improved accordingly, which makes the fiber reinforcement achieve the best. The stress–strain relationship is approximately hyperbolic and strain hardening. The characteristics of strain hardening are more obvious with the increase in matric suction, and the soil specimens present plastic failure. The volumetric strain of specimen is more sensitive to the changing net confining pressure. It increases with the increase in net confining pressure, and increases linearly as the matric suction is zero. The failure modes of triaxial tests are divided into tensile failure and friction failure.

## 1. Introduction

Unsaturated soils are widely distributed in nature. The upper soil layer is almost unsaturated in human activities and engineering construction. Due to the matric suction, unsaturated soils are more complex and variable than saturated soils [1]. Many scholars regard matric suction as the focus of unsaturated soil properties [2,3,4]. The development of new economic and effective soil improvement technology has been a research hotspot in the fields of geotechnical, geological, and road engineering. The fiber reinforced soil improvement technology is one of the most commonly used technologies. Polypropylene fiber can be evenly distributed in soil, which obviously enhances the mechanical properties of the soil, and improves the shear strength and permeability [5,6,7].

A lot of experimental studies have been carried out by scholars on shear strength of unsaturated soils and polypropylene fiber reinforced soils. Some important theories and experiences are summarized. Lashkari [8] establishes the structural interface model of soils with dry/saturated state and extends to the unsaturated soil state by studying the effective stress, constitutive equation of the critical state. Six basic cases are selected to analyze the effects of matric suction and net vertical stress on peak and residual shear strength, the volumetric change behavior of unsaturated soils. A constitutive model for simulating the behavior of unsaturated interfaces is presented by Lashkari [9]. In addition, an extended model is formulated in terms of two pairs of work conjugate stress–strain-like variables. For each interface type, it is shown that the proposed model can capture the essential elements of the behavior using a unique set of parameters. In order to effectively solve the problem of stability and bearing capacity of unsaturated soil slope, Chiorean [10] simulated the boundary conditions of rainfall, evaporation, and equilibrium to predict the change in matric suction in soil according to the nonlinear characteristics of the soil–water characteristic curves and the seepage phenomenon of groundwater, respectively. According to the drying and wetting paths of soil–water characteristic curves, Chaminda [11] improves a direct shear device, and measures the shear strength of silty clay under low matric suction. The results show that the matric suction and water characteristic curves of drying and wetting soil have little effect on the internal friction angle, and the initial stiffness and peak stress increase with the increase in matric suction; Liu [12] uses the two-chamber triaxial system (DCTS) to conduct a triaxial test with the particle fill of the river bank to determine its critical state parameters; Abd [13] studies the shear strength of unsaturated soils with changing matric suction, and finds that the relationship between shear strength and matric suction is linear or nonlinear, and the maximum shear stress appears at high matric suction; and Gao [14] studies the change in hydraulic properties of unsaturated cohesive silt at high matric suction.

Taha [15] conducts shear, consolidation, and microstructure tests on different polypropylene fiber mixed cohesive soils. The results show that the mechanical properties improve with polypropylene fiber adding into the cohesive soils, and the optimal fiber dosage is 3%. Clay is sensitive to the change in moisture in the soil. In some cold climates, freeze–thaw cycles occur in the soil. Scholars have studied the soil properties of polypropylene fiber reinforced clay after freeze–thaw cycles. Parameters of soil properties, such as size change rate, stress–strain curves, and post-peak stress ratio, can be obtained [16,17,18]. Mirzababaei [19] makes a series of multi-stage reverse drainage direct shear tests to study the shear strength of polypropylene fiber reinforced clay with different initial pore ratios under large shear displacement. The results show that the fiber reinforcement on the effective stress ratio decreases with the increase in effective stress and initial pore ratio. The optimal fiber dosage is 0.25%. It is related to the initial pore ratio rather than the stress history and effective stress.

The research findings of unsaturated soils and fiber reinforced soils are shown above from different angles. The shear properties of unsaturated soils and fiber reinforcement technology are very important in engineering practice, but it needs to be pointed out that the influence of matric suction on unsaturated soils with polypropylene fiber reinforcement is less considered, and the reinforcement mechanism of polypropylene fiber for unsaturated soils has not yet formed a unified understanding.

Therefore, polypropylene fiber is used as reinforcement material, and unsaturated clay in Shaoxing section of East Zhejiang Grand Canal is selected in the paper. The paper focuses on the influence of matric suction on the shear properties of polypropylene fiber reinforced unsaturated clay with changing net confining pressure by triaxial apparatus for unsaturated soils, which provide a theoretical basis for engineering practice.

## 2. Test Materials and Scheme

### 2.1. Test Materials

**Figure 1:**
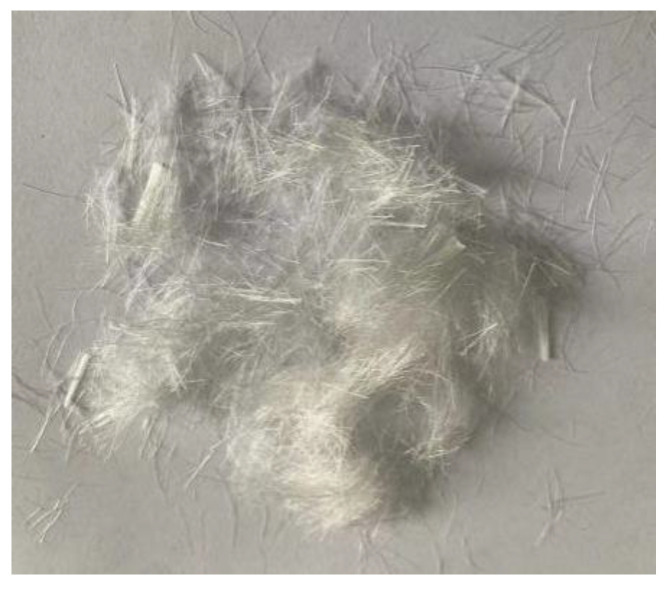
Polypropylene fibers.

The soil reinforcement material is polypropylene fiber, which is a polymer compound made of propylene (chemical formula is CH_3_-CH=CH_2_). It is a well-structured crystalline polymer with strong acid and alkali resistance and good chemical stability. Its surface is hydrophobic, non-absorbent, and non-toxic, with high tensile strength and low elastic modulus. It can be evenly distributed in soils, and produces a good grip with soil particles [20]. The polypropylene fiber is shown in Figure 1, and the basic physical and mechanical parameters are shown in Table 1.

**Figure 2:**
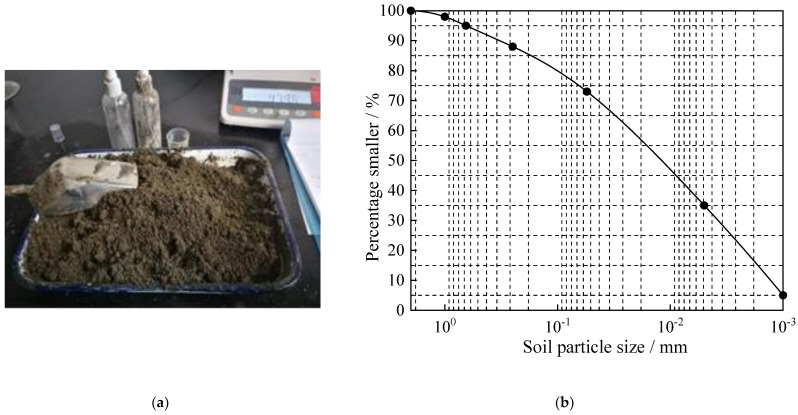
Soil specimen and grain distribution curve. (**a**) Soil specimen, (**b**) particle size distribution curves.

The test soil specimen was taken from a foundation pit in Shaoxing section of East Zhejiang Grand Canal, and the soil depth was 9.0–11.0 m. It was upper Pleistocene clayey soil in Tertiary, and the sedimentary origin of early Holocene fluvial-lacustrine facies and delta transitional facies. It was brown and yellow-gray, plastic, soft-plastic, local fluid-plastic, and medium-high compression, with a small amount of Fe-Mn contamination and calcareous nodules [21]. Figure 2 shows the distribution curves of test soil specimens and particles, respectively.

The undisturbed soils were put into the oven in laboratory, and dried for 24 h at 106 °C, crushed, sieved through 2 mm, and then, the natural stacking quartering method was used for diagonal sampling. The preparation method for polypropylene fiber reinforced soil is proposed by Sun [22]. According to the test scheme, test materials included dry soil, polypropylene fiber, and airless distilled water. The first prepared soil specimen was prepared by weighing 1/3 portion of Test materials, and then 1/3 portion of test material was mixed into the first prepared soil specimen in the same way to obtain the second prepared soil specimen. In this way, the fiber reinforced soils were prepared three times in total to ensure the uniformity of polypropylene fibers in the soil specimens. After the soil specimen with the target water content was prepared, the mixed soil specimen was put into a sealed plastic bag and maintained in a moisturizer for 24 h, so that the water distribution in the soil specimen was uniform. After 24 h, the water content of the reinforced soil specimen was remeasured. If the water content difference between the soil specimen and the target specimen was not more than 1%, it met the test requirements. If the difference exceeded 1%, the soil specimen was reprepared. The physical properties of soil specimen are shown in Table 2.

**Figure 3:**
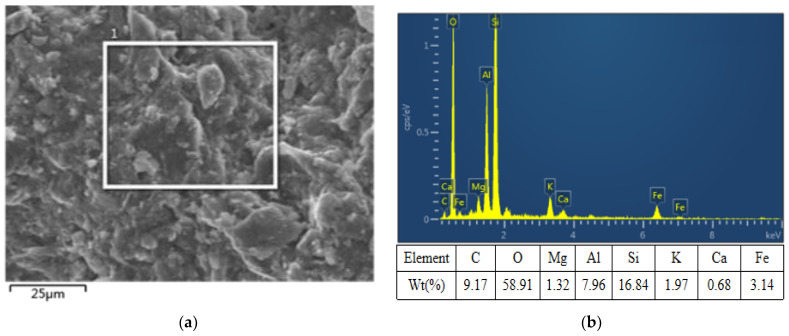
SEM-EDS analysis of a soil specimen: (**a**) SEM and (**b**) EDS.

**Figure 4:**
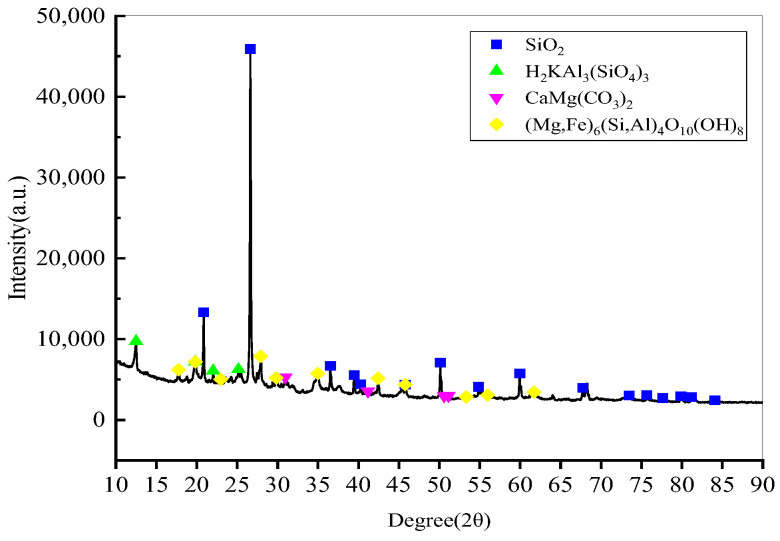
XRD spectrum of a soil specimen.

The soil specimens were tested by X-Ray Diffraction(XRD) and Energy Dispersive Spectroscopy(EDS) for mineralogical analysis. Scanning Electron Microscope (SEM) scanning electron microscope adopts Nissan high–low vacuum scanning electron microscope (JSM-6360LV, JEOL, Tokyo, Japan). The model of XRD electron ray diffractometer was Empyrean(PANalytical, Almelo, Netherlands). The morphologies of a soil specimen presented observed by SEM is displayed in Figure 3a. EDS analyses were conducted aiming to elucidate the elemental compositions of a soil specimen(JSM-6360LV, JEOL, Tokyo, Japan), as is shown in Figure 3b. The results show that the main elemental compositions of a soil specimen were O, Si, C, and Al. XRD spectra of a soil specimen are presented in Figure 4. As can be seen from the Figure, there are four main crystalline phases in a soil specimen. SiO_2_ has a strong diffraction peak at 26.64° with high intensity and narrow half-peak width. In short, the results of mineralogical analysis with EDS and XRD are in good agreement.

### 2.2. Test Scheme

Based on the axes transmission technology, the consolidated drained shear test for unsaturated soils were carried out with changing matric suction and net confining pressure. The initial water content was 25% and the dry density was 1.5 g/cm^3^. According to the research results of other scholars [23], the matric suction range that affects shear strength for unsaturated soils was usually 0 to 500 kPa. Therefore, in this study, the matric suction was taken as 0, 50 kPa, 100 kPa, 200 kPa, 300 kPa, and the net confining pressure of the test under the same matric suction was as follows: 50 kPa, 100 kPa, 200 kPa. The fiber dosage was 0.2%, that is, the mass ratio of fiber and dry soil was 0.2%. The fiber length was 6 mm, 12 mm, 19 mm, and the fiber diameter was 0.04 mm, so the change in fiber length was the change in fiber aspect ratio. In the test, the shear rate was 0.002 mm/min. The fiber dosage was 0.2%, with lengths of 6 mm, 12 mm, and 19 mm. The shear rate was set to 0.002 mm/min. We stopped testing when shear strain reached 15%. If the stress vs. strain curve has an obvious peak, the peak value is the failure deviatoric stress. If there is no peak, the value corresponding to the strain of 15% is the failure deviatoric stress. The standard to judge the equilibrium of consolidation and suction stage is that the volume change and drainage are less than 0.01 cm^3^ within 2 h, and the minimum duration is 40 h. In this experimental study, the fiber dosage was small, and the specimen preparation process was carried out in strict accordance with the relevant specifications, so the phenomenon of the balling effect in the soil is less, which should be ignored in the analysis of the test results.

### 2.3. Test Process

The weight of soil is determined considering the target dry density. Cylinder trivalve is used to prepare soil specimens. First, a small amount of Vaseline was smeared to the inner wall of the cylinder trivalve. Second, the soil was evenly divided into five layers and compacted, and each layer was compacted to the required height, the surface was made coarse, so as to strengthen the uniformity of fiber in the soil and ensure the quality of the specimen. Finally, the specimen was placed in a vacuum saturator to exhaust air, lasting for 24 h to ensure full saturation [24]. The standard size of the triaxial soil specimen was 39.1 mm × 80 mm. In the test, the length effect of fibers was considered mainly, and the bending effect of a small amount of fibers was ignored.

The “no air” water was prepared before the test. The air in the pipeline and controller of the apparatus needed to be cleaned, and the ceramic plate was saturated for more than 24 h. The soil specimen was taken out of the vacuum saturator and weighed. On one end of the specimen, a permeable stone and a piece of filter paper were put on, and on the other end, a piece of filter paper on the ceramic plate without permeable stone. The specimen was fixed with a rubber film. Internal and external pressure chamber covers were installed, and the axial pressure sensor was tightly placed on the specimen cap. We checked whether there was water leakage or air leakage after injecting water and exhausting the air. Test preparation was made ready by opening or closing the corresponding valve.

### 2.4. Test Instrument

**Figure 5:**
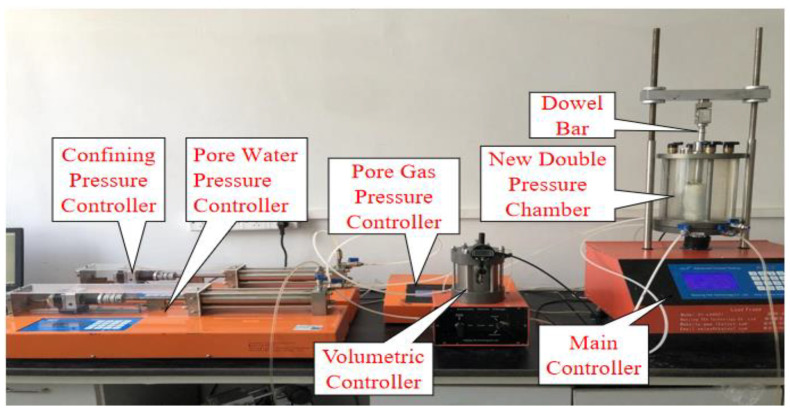
TKA double pressure triaxial apparatus for unsaturated soils.

The TKA triaxial apparatus with a double-pressure chamber for unsaturated soils in this test was made in Nanjing Tekeao Company. The internal and external pressure chamber walls are composed of high strength plexiglass, which can completely remove water adsorption and effectively reduce the error. The controller and data acquisition of the instrument are all controlled automatically by the computer. The apparatus consists of a double-pressure chamber, controlling system, measurement and data acquisition system, and data processing, as is shown in Figure 5.

## 3. Test Results and Analysis

### 3.1. Test Data Processing

The test results are calculated by Fredlund bivariate shear strength formula for unsaturated soils [25]:

(1) $$\tau_{f}=c^{\prime }+\left(\sigma -u_{a}\right)\tan\varphi^{\prime }+\left(u_{a}-u_{w}\right)\tan\varphi^{b}$$

where *c*′ is the effective cohesion intercept; *σ* and *u*_a_ are the total vertical stress and the pore air pressure, so (*σ* − *u*_a_) is the net vertical stress; *u*_w_ is the pore water pressure; *φ*′ and *φ^b^* are the effective internal friction and the adsorption internal friction angle.

The shear strength formula for unsaturated soils can also be written as follows [24]:

(2) $$\tau_{f}=c+\left(\sigma -u_{a}\right)\tan\varphi^{\prime }$$

in which, $c=c^{\prime }+\left(u_{a}-u_{w}\right)\tan\varphi^{b}$, *c* is the cohesion intercept obtained by the test including effect of matric suction, and is called total cohesion intercept. By combining Equation (2) with *c*, we obtain Equation (1). According to the three-dimensional failure envelope diagram for unsaturated soils, the linear intercept of the *τ*_f_ − (*σ* − *u*_a_) plane is the total cohesion intercept, and the slope is the effective internal friction angle. The values are obtained by the test, and *c*′ is obtained by triaxial test of saturated soils. Thus, the value of *φ^b^* can be obtained. The value of *φ^b^* is equal to *φ*′ when the matric suction is zero (saturated soils). The values obtained by the test are shown in Table 3.

### 3.2. Effects of Matric Suction on the Shear Properties of the Specimens

**Figure 6:**
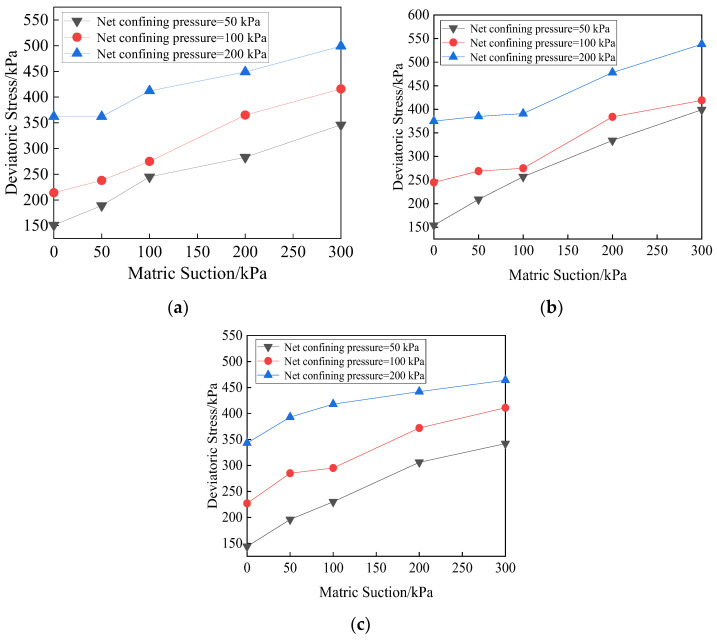
Relationships between matric suction and deviatoric stress with different lengths of fibers. (**a**) Fiber length = 6 mm, (**b**) Fiber length = 12 mm, (**c**) Fiber length = 19 mm.

The shear properties for unsaturated soils are comprehensive reflection of the mechanical action of the internal structure inside the soil, which are related to several factors, such as matric suction, cementation characteristics between particles, molecular gravitation between particles, physical and chemical action of water membrane, and electrostatic force. The change in matric suction will cause the change in water–air shrinkage membrane inside the soil, and then change the soil properties, especially the shear strength. The effect of matric suction on deviatoric stress for the specimens with different lengths of fiber is shown in Figure 6.

**Figure 7:**
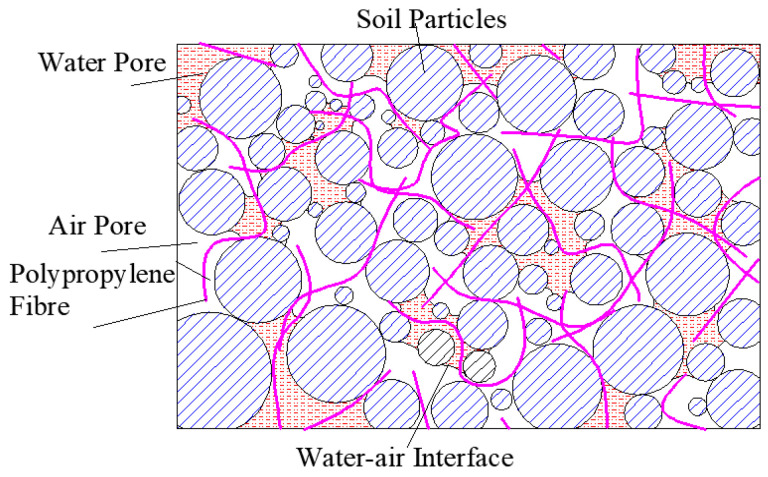
Schematic diagram of microstructure for fiber reinforced soil specimen.

As can be seen from Figure 5, the greater the matric suction, the greater the net confining pressure and the deviatoric stress are. When the net confining pressure is small, the deviatoric stress increases linearly with the increase in the matric suction. When the net confining pressure is large, the increase in deviatoric stress of matric suction with short fiber reinforced specimen in the range of 100–200 kPa is greater than that in the range of 0–100 kPa. When the fiber length is 12 mm and the net confining pressure is 50 kPa, the deviatoric stress increases significantly, and it reaches 245 kPa. The matric suction has a great influence on the shear properties when the net confining pressure is small. The reason is that the effective contact surface between fibers and soil particles and among soil particles is small, a lot of free water–air interface in soil will form a meniscus water membrane, which produces matric suction affecting soil properties. The change in matric suction is directly related to the number of shrinkage membranes in soil, so the soil strength is sensitive to the change in matric suction. When the net confining pressure is large, the soil particles are arranged densely, the friction between fibers and soil particles is large, and the matric suction has little effect on the shear strength of the soil. At this time, the fibers in soil are the main factor affecting the strength. The microstructure of the soil specimen is observed by electron microscope, and it is described by sketch, as shown in Figure 7.

**Figure 8:**
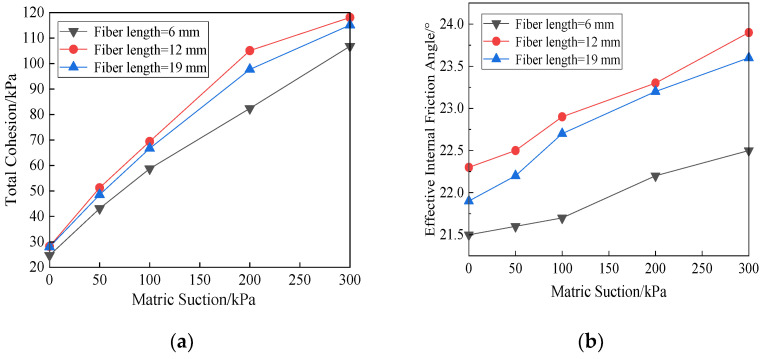

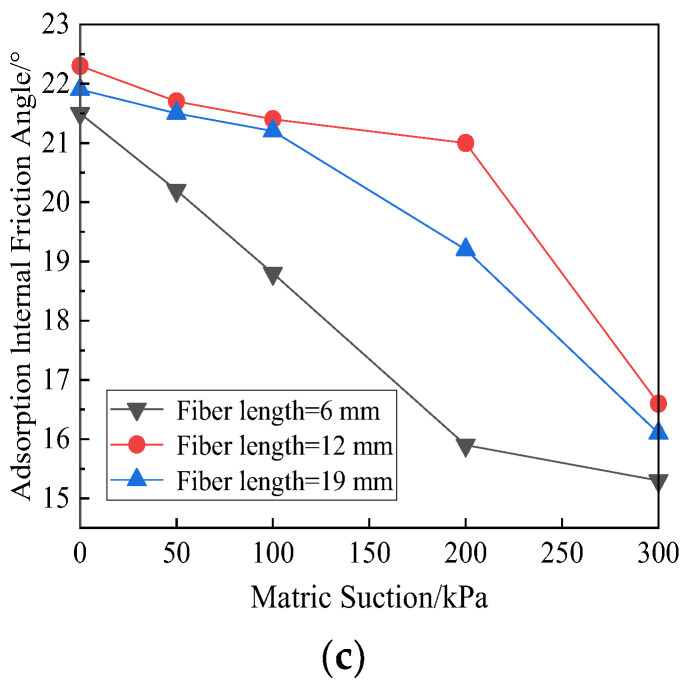
Influence of matric suction on shear strength parameters. (**a**) Effects of the matric suction on the total cohesion force, (**b**) effects of matric suction on the effective internal friction angle, (**c**) effects of the matric suction on the adsorption internal friction angle.

The effect of matric suction on the shear strength parameters of the soil specimens with different fiber lengths is shown in Figure 8. It can be seen that the total cohesion intercept and the effective internal friction angle of the soil specimen increase with the increase in matric suction. As the fiber dosage is fixed and the fiber length is 12 mm, the shear strength parameters of the soil specimens achieve the best, that is, the shear strength of the soil specimen is the maximum under the same conditions. When the fiber length is 12 mm, the total cohesion intercept increases by 89.9 kPa and the effective internal friction angle by 1.6° during the matric suction ranging from 0 to 300 kPa. The increase in the total cohesion intercept decreases when the matric suction exceeds 200 kPa. Therefore, the effect of matric suction on total cohesion intercept is greater than effective internal friction angle for the polypropylene fiber reinforced unsaturated clay. With the increase in matric suction, the adsorption internal friction angle decreases gradually. When the fiber length is 12 mm and the matric suction ranges from 200 kPa to 300 kPa, the adsorption internal friction angle decreases by 4.4°. The adsorption internal friction angle reflects the increasing rate of shear strength with matric suction. It indicates that the greater the matric suction is, the smaller contribution of matric suction to the increase in soil shear strength is.

Polypropylene fibers form a three-dimensional mesh structure in soil. The fibers and soil particles act together. When the matric suction is small, a small amount of air exists in the corners of individual pores, and the shear strength increases slightly. With the increase in matric suction, soil particles lack water lubrication. Because of the influence of polypropylene fibers, the thickness of the shrinkage membrane of water–air interface decreases, which increases the connection between soil particles and the frictional resistance between fibers and soil particles. When the matric suction is large enough, the proportion of pore water decreases, and the number of shrinkage membrane also decreases. The number of discontinuous water–air interface shrinkages at the membrane is the microscopic enhancing mechanism of matric suction to shear strength, so the adsorption internal friction angle decreases gradually.

### 3.3. Effects of Matric Suction on Stress vs. Strain Curves

**Figure 9:**
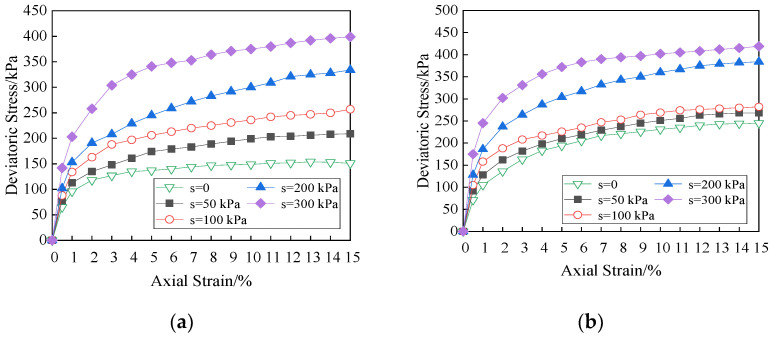

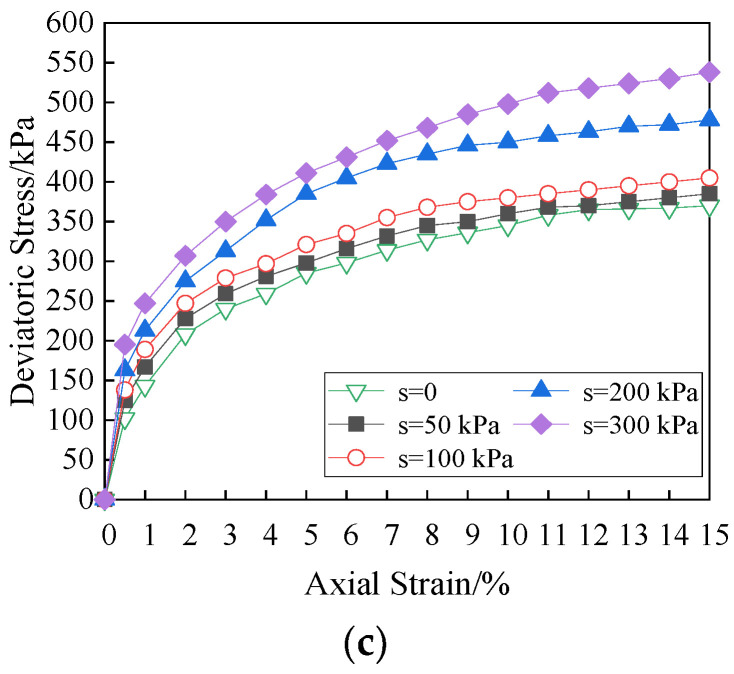
Effects of matric suction on stress vs. strain curves (Fiber length = 12 mm). (**a**) Net confining pressure = 50 kPa, (**b**) net confining pressure = 100 kPa, (**c**) net confining pressure = 200 kPa.

The triaxial test is a common laboratory way to determine stress–strain relationship of the soil, which can truly reflect the actual loading condition, and the stress state and drainage condition are clear. Under different net confining pressures, the effects of matric suction on the stress vs. strain curves for soil specimen at 12 mm fiber is shown in Figure 9, in which “*s*” is matric suction.

As can be seen from the figure, under the action of matric suction, the stress vs. strain curves of fiber reinforced unsaturated clay are approximately hyperbolic, and they are all strain hardening. The greater the matric suction is, the more obvious the strain hardening is, and the soil specimen presents plastic failure. Under the cohesion and the internal friction, the fibers bear part of the tensile stress of the soil specimens deformation, and inhibit effectively the development of cracks in soil. The axial strain is within 3%. The growth rate of deviatoric stress is large when the curve slope is large. The curve slope and the growth rate of deviatoric stress decreases gradually with the axial stress exceeding 3%. With the increase in axial strain, the smaller the net confining pressure and matric suction are, the smaller the curve slope is. At the beginning of shear process, there is no obvious difference in the curve slope due to the influence of matric suction, and the initial stiffness of the fiber reinforced soils is similar. It indicates that the contribution of the matric suction to the shear strength is not obvious. With increasing axial strain, the soil particles rearrange and the fibers bend under compression, and the effect of matric suction is more and more obvious.

**Figure 10:**
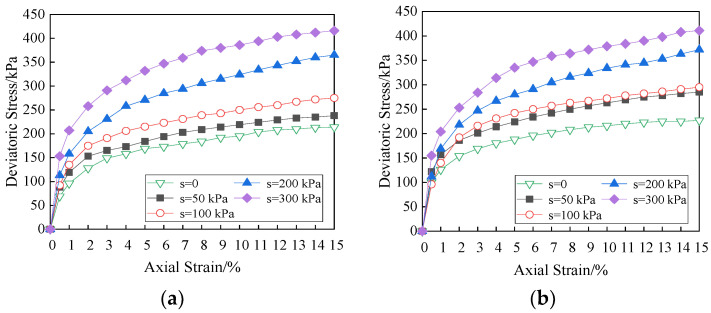
Effects of matric suction on stress vs. strain curves (Net confining pressure = 100 kPa). (**a**) Fiber length = 6 mm, (**b**) fiber length = 19 mm.

It is the demarcation point that the value of matric suction is 100 kPa. The change trend of stress-strain relationship with the increase in matric suction shows polarization. It is obvious with increasing net confining pressure. When fiber dosage is 0.2% and the fiber length is 12 mm, the fibers and the pores distribute uniformly in the soil, and more continuous concave surface is formed. The matric suction is produced by the water–air shrinkage membrane. Therefore, the matric suction has an obvious influence on the deviatoric stress. Figure 10 is the stress vs. strain curves with the fiber length of 6 mm and 19 mm under the net confining pressure of 100 kPa, and the influence of matric suction is consistent with the above analysis.

### 3.4. Effects of Matric Suction on the Volumetric-Strain vs. Axial-Strain Curves

The triaxial apparatus with a double-pressure chamber for unsaturated soils is used in this test. Because the chamber walls are made of high strength Perspex, and they bear the same confining pressure, their deformation can be ignored. Therefore, the water volume change in the internal pressure chamber is equal to the volume change in the soil specimen, and the volume change can be automatically and continuously recorded by the volume change meter.

The relationship between volumetric strain and axial strain of the soil specimen with fiber length of 12 mm in the shearing process is shown in Figure 10. The negative volumetric strain indicates contraction, and the positive volumetric strain indicates dilation. As can be seen from the figure, under different net confining pressures, the smaller the matric suction is, the larger the volumetric strain is. The growth trend of volumetric strain with matric suction of 0 is the most obvious, showing linear growth. When the net confining pressure is 200 kPa, the specimen with matric suction of 50 kPa shows a transient contraction at the early stage and then turned to dilation, and the specimen shows dilatancy during the shear process. The matric suction increases from 0 to 300 kPa when the net confining pressure is 50 kPa, 100 kPa, and 200 kPa, respectively, and corresponding volumetric strain decreases by 48%, 0.60%, and 0.61%, respectively. Therefore, the larger the net confining pressure is, the larger volume reduction is. When the net confining pressure is 200 kPa, at the beginning of shear, the increase in volumetric strain of the specimen under smaller matric suction is significantly greater than that under larger matric suction. After the axial strain reaches 4%, the increase in volumetric strain decreases gradually.

**Figure 11:**
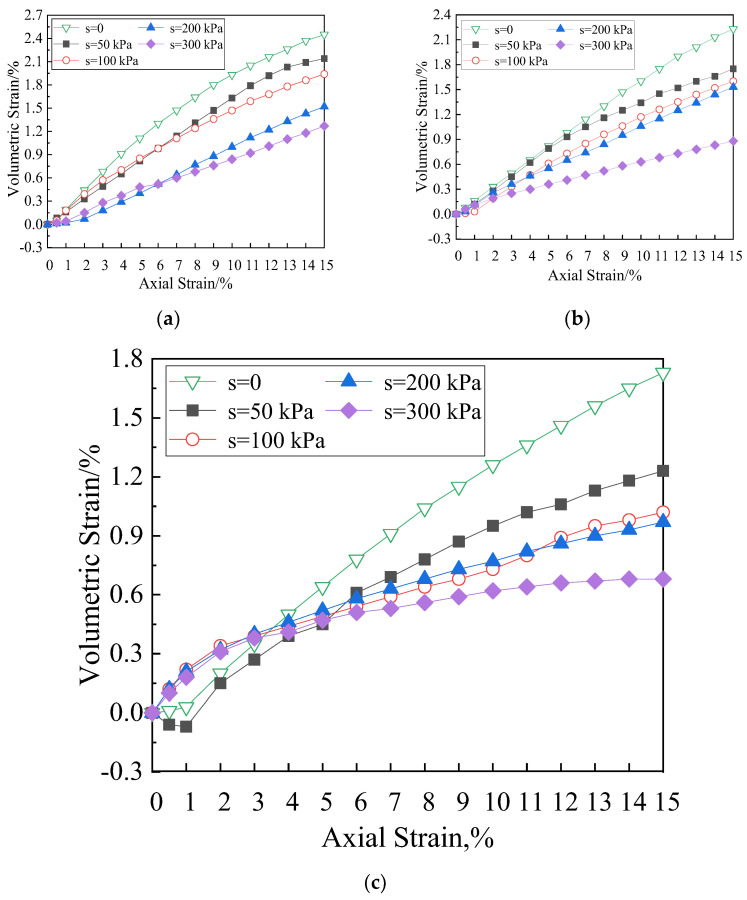
Effects of matric suction on volumetric strain vs. axial strain curves (Fiber length = 12 mm). (**a**) Net confining pressure = 50 kPa, (**b**) net confining pressure = 100 kPa, (**c**) net confining pressure = 200 kPa.

**Figure 12:**
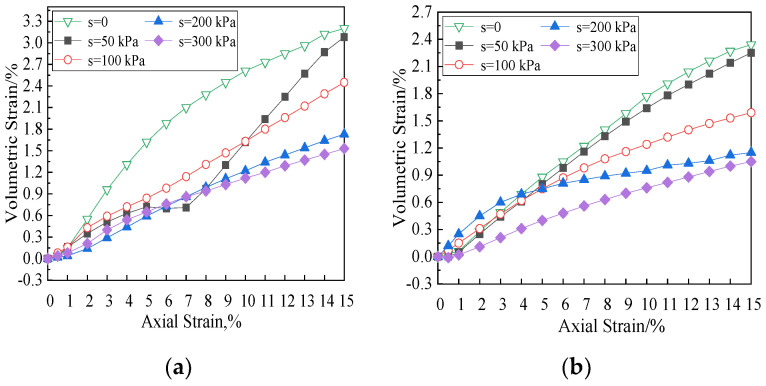
Effects of matric suction on volumetric strain vs. axial strain curves (net confining pressure = 100 kPa). (**a**) Fiber length = 6 mm, (**b**) fiber length = 19 mm.

Under different net confining pressures, the volumetric strain is affected obviously by the matric suction. The smaller the matric suction is, the larger the volumetric strain and the curve slope (Figure 11) are. This phenomenon is mainly due to the fact that there are only polypropylene fibers, soil particles, and pore water in saturated soils (matric suction is 0), and polypropylene fibers and soil particles are incompressible. The volume change in the specimen is completely caused by the discharge of pore water. Therefore, the volumetric strain of the specimen is the largest when the matric suction is 0. When the matric suction increases, the proportion of pore air in the specimen increases too. In the triaxial consolidation with equal suction, the volume of the specimen decreases, and the pore in the soil decreases, so the soil mass is compacted. Therefore, with the increase in net confining pressure during shear process, the smaller the matric suction is, the larger the volumetric strain is. Polypropylene fibers can effectively restrain the deformation of soil mass and displacement of the soil particles, improve the antideformation ability, and delay the development of cracks. The results show that the fiber reinforced unsaturated soils have plastic failure characteristics and large residual strength. Figure 12 shows the influence of matric suction on the volumetric strain of the specimen at 6 mm and 19 mm fiber length with the net confining pressure of 100 kPa, and the variation of the curves conform to the above analysis.

**Figure 13:**
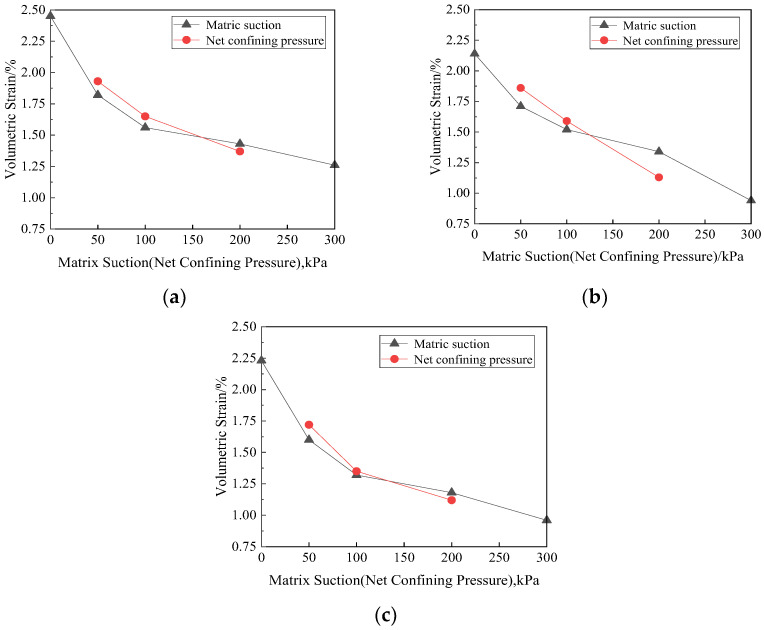
Relationships of matric suction or net confining pressure and volumetric strain. (**a**) Fiber length = 6 mm, (**b**) fiber length = 12 mm, (**c**) fiber length = 19 mm.

**Figure 14:**
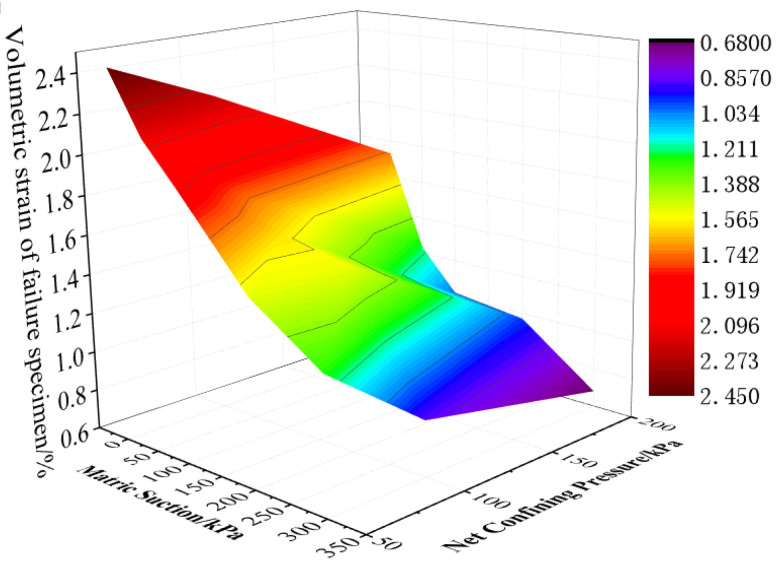
Cloud diagram of volumetric strain with matric suction and net confining pressure.

The volumetric strain of the failure specimens with the same matric suction and net confining pressure is taken as the average value to plot the curve, as shown in Figure 13. As can be seen from the figure, when the net confining pressure is constant, the volumetric strain of the specimen decreases greatly whether the matric suction is large (greater than 100 kPa) or small (less than 50 kPa), but it has little decrease in the range of 50–100 kPa. The volumetric strain decreases with the increase in matric suction and net confining pressure, but it varies flat with matric suction relatively. According to the generalized suction principle proposed by Sun [26], the anti-deformation ability does not increase proportionally with the increase in matric suction, which indicates that part of the suction can increase the strength and anti-deformation ability. The volumetric strain of the specimen is more sensitive to the change in net confining pressure. Figure 14 is cloud diagram of volumetric strain with matric suction and net confining pressure. In short, the change trend of volumetric strain with matric suction and net confining pressure during the failing process of specimens can be intuitively seen.

### 3.5. Analysis of Triaxial Specimens

**Figure 15:**
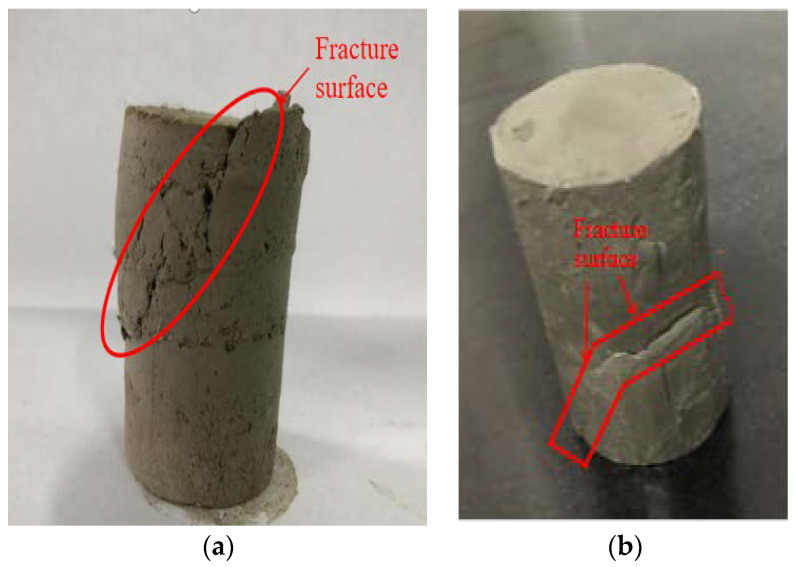
Tensile failure specimen. (**a**) Fiber reinforced specimen, (**b**) specimen without polypropylene fiber.

According to the existing research results, there are two main failure modes of fiber reinforced soil triaxial specimens: tensile failure and friction failure. Figure 14 and Figure 15 show the tensile failure and friction failure of polypropylene fiber reinforced soil sample and soil sample without fiber, respectively.

1. Tensile failure: the soil specimen has obvious fracture surface, and the fibers manifest mainly as tensile fracture during shear process. The main reason is that the polypropylene fibers are fractured due to the large friction resistance as the axial displacement reaches large enough. Fibers can effectively provide tensile stress to hinder the development of cracks, thus enhancing the shear strength.
2. Friction failure: There is no obvious fracture surface in the shear failure of the soil specimen, and the volume change shows a radial drum trend. The fibers in the soil specimen have no obvious fracture. The insufficient friction between fibers and soil particles leads to the slippage, and the soil specimen represents compression deformation. It shows that the fiber reinforced soil has the characteristics of approximate isotropy, and the fibers form a spatial network structure in the soil, which plays a role of disorderly support and makes the soil stress more uniform.

During shear process, polypropylene fibers are anchored in the soil, not easy to pull out, and bear a certain tensile stress, thus forming a three-dimensional network structure in the soil. The larger the net confining pressure is, the larger the effective area between fibers and surrounding soil particles is. These lead to enhancing interface friction of fibers and soil particles. The greater the matric suction is, the smaller the contribution of matric suction to the shear strength is. From the above analysis of the influence of the matric suction and the net confining pressure on the volumetric strain, we obtain the conclusions that soil specimens are prone to tensile failure as net confining pressure is large, and friction failure as matric suction is large.

**Figure 16:**
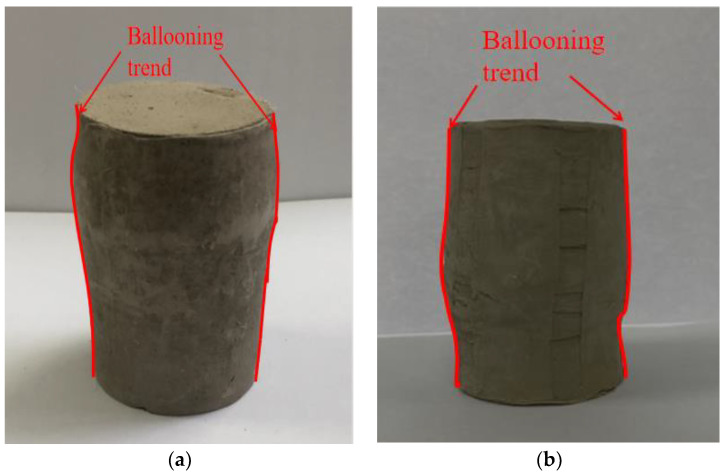
Friction failure specimen. (**a**) Fiber reinforced specimen, (**b**) specimen without polypropylene fiber.

It can be found from Figure 15 that the fracture surface of the fiber-reinforced soil is relatively rough, where the soil particles form small groups and interact with the fibers, and many irregular cracks develop on the fracture surface. During shear process, the fibers form a three-dimensional network structure and soil particles act each other, which can lead the fiber pull part of the soil particles away from the main body at the fracture surface. As the soil without fiber is sheared, the shear plane is relatively smooth. It can be seen from Figure 16 that the friction failure develops regularly in the surrounding direction of the fiber reinforced soil specimen, while the friction failure presents irregular swelling in the surrounding direction of the soil specimen without fibers. The polypropylene fibers’ disorderly support-effect makes the stress in soil more uniform, which can effectively improve the shear strength of soil.

## 4. Conclusions

1. The greater the matric suction and the net confining pressure are, the greater the deviatoric stress of the specimen is. When the net confining pressure is small, the matric suction has obvious effects on the shear strength of the soil. The total cohesion intercept and effective internal friction increase with the increase in matric suction, while the adsorption internal friction angle decreases gradually. Similarly, the contribution of matric suction to shear strength decreases. The total cohesion intercept is more sensitive to matric suction. Under the same conditions, as the fiber length is 12 mm, the shear strength is the largest, which means that the reinforcement effect of polypropylene fiber achieves the best.
2. The stress–strain relationship is approximately hyperbolic and strain hardening. The characteristics of strain hardening are more obvious with the increase in matric suction, and the soil specimens present plastic failure. The effect of matric suction on the shear strength is obvious with increasing axial strain.
3. With the increase in net confining pressure during shearing process, the matric suction is smaller, and the volumetric strain is larger. The volumetric strain of specimen is more sensitive to the changing net confining pressure. It increases with the increase in net confining pressure. The relations present linearly as the matric suction is 0. Soil specimens are prone to tensile failure as net confining pressure is large, and friction failure as matric suction is large.

## Figures and Tables

**Table 1:** Physical and mechanical properties of polypropylene fiber.

Type	Density /(g·cm^−3^)	Diameter /mm	Strength of Extension /MPa	Elasticity Modulus /MPa	Light /°C	Acid-Base Resistance Property	Dispersity
Bunchy Monofilament	0.91	0.04	>486	>4800	590	Pole-strength	Excellent

**Table 2:** Physical properties of soil specimen.

Liquid Limit *ω*_L_/%	Plastic Limit *ω*_P_/%	Plasticity Index *I*_P_/%	Relative Density d_s_	Maximum Dry Density *ρ*_dmax_/(g·cm^−3^)	Optimal Water Content *ω*_op_/%	Grain Composion (mm)&Dosage(%)		
						Clay <0.005	Silt 0.005~0.075	Sand >0.075
42.7%	23.3%	19.4%	2.72	1.69	23.7%	39.80	47.32	12.88

**Table 3:** Shear characteristics of fiber reinforced unsaturated soils.

Fiber Length /mm	Matric Suction/kPa	Net Confining Pressure /kPa	Deviatoric Stress /kPa	Total Cohesion /kPa	Effective Internal Friction Angle/°	Adsorption Internal Friction Angle/°
6	0	50	151	24.7	21.5	21.5
		100	214			
		200	362			
	50	50	189	43.1	21.6	20.2
		100	238			
		200	362			
	100	50	245	58.7	21.7	18.8
		100	275			
		200	412			
	200	50	283	82.4	22.2	15.9
		100	365			
		200	449			
	300	50	346	106.8	22.5	15.3
		100	416			
		200	499			
12	0	50	154	28.2	22.3	22.3
		100	245			
		200	375			
	50	50	209	51.2	22.5	21.7
		100	269			
		200	385			
	100	50	257	69.4	22.9	21.4
		100	275			
		200	391			
	200	50	334	105.1	23.3	21
		100	384			
		200	478			
	300	50	399	118.1	23.9	16.6
		100	419			
		200	538			
19	0	50	144	28.0	21.9	21.9
		100	227			
		200	343			
	50	50	196	48.5	22.2	21.5
		100	285			
		200	393			
	100	50	230	66.7	22.7	21.2
		100	295			
		200	418			
	200	50	306	97.7	23.2	19.2
		100	372			
		200	442			
	300	50	342	115.1	23.6	16.1
		100	411			
		200	464			

## Data Availability

Not applicable.
